# Interleukin-6 and granulocyte colony-stimulating factor as predictors of the prognosis of influenza-associated pneumonia

**DOI:** 10.1186/s12879-022-07321-6

**Published:** 2022-04-06

**Authors:** Jiaying Zhang, Jingxia Wang, Yiwen Gong, Yudan Gu, Qiangqiang Xiang, Ling-Ling Tang

**Affiliations:** 1 State Key Laboratory for Diagnosis and Treatment of Infectious Diseases, National Clinical Research Center for Infectious Diseases, Collaborative Innovation Center for Diagnosis and Treatment of Infectious Diseases, The First Affiliated Hospital, College of Medicine, Zhejiang University, Hangzhou, 310003 People’s Republic of China; 2 Shulan (Hangzhou) Hospital Affiliated to Zhejiang Shuren University, Shulan International Medical College, Hangzhou, 310000 People’s Republic of China; 3 Zhejiang Hospital of Integrated Traditional Chinese and Western Medicine, Hangzhou, 310000 People’s Republic of China; 4 Zhejiang Chinese Medical University, Hangzhou, 310000 China

**Keywords:** Influenza, Pneumonia, Cytokine, Interleukin-6, Granulocyte colony-stimulating factor, Neutrophil

## Abstract

**Background:**

Pneumonia is a common complication of influenza and closely related to mortality in influenza patients. The present study examines cytokines as predictors of the prognosis of influenza-associated pneumonia.

**Methods:**

This study included 101 inpatients with influenza (64 pneumonia and 37 non-pneumonia patients). 48 cytokines were detected in the serum samples of the patients and the clinical characteristics were analyzed. The correlation between them was analyzed to identify predictive biomarkers for the prognosis of influenza-associated pneumonia.

**Results:**

Seventeen patients had poor prognosis and developed pneumonia. Among patients with influenza-associated pneumonia, the levels of 8 cytokines were significantly higher in those who had a poor prognosis: interleukin-6 (IL-6), interferon-γ (IFN-γ), granulocyte colony-stimulating factor (G-CSF), monocyte colony-stimulating factor (M-CSF), monocyte chemoattractant protein-1 (MCP-1), monocyte chemoattractant protein-3, Interleukin-2 receptor subunit alpha and Hepatocyte growth factor. Correlation analysis showed that the IL-6, G-CSF, M-CSF, IFN-γ, and MCP-1 levels had positive correlations with the severity of pneumonia. IL-6 and G-CSF showed a strong and positive correlation with poor prognosis in influenza-associated pneumonia patients. The combined effect of the two cytokines resulted in the largest area (0.926) under the receiver-operating characteristic curve.

**Conclusion:**

The results indicate that the probability of poor prognosis in influenza patients with pneumonia is significantly increased. IL-6, G-CSF, M-CSF, IFN-γ, and MCP-1 levels had a positive correlation with the severity of pneumonia. Importantly, IL-6 and G-CSF were identified as significant predictors of the severity of influenza-associated pneumonia.

**Supplementary Information:**

The online version contains supplementary material available at 10.1186/s12879-022-07321-6.

## Introduction

Influenza is common detected in respiratory infection that can spread rapidly through communities. Throughout history, influenza pandemics have killed tens of thousands of people [1]. Globally, seasonal influenza causes significant morbidity and mortality. According to the World Health Organization (WHO), 3 to 5 million people suffer from the flu each year, and nearly 10% of the patients die as a result [2]. Pneumonia is an important complication of influenza. Studies have shown that influenza-associated pneumonia is an independent mortality-related factor in cases of influenza [3]. Therefore, the early prediction of the severity of influenza-associated pneumonia is of great significance with regard to reducing the mortality of seasonal influenza. However, biomarkers for disease severity and progress are lacking. The identification of reliable biomarkers of prognosis would help in the prevention and timely treatment of pneumonia in influenza patients.

Cytokines are a diverse group of small proteins that regulate immune and inflammatory responses [4]. A cytokine storm is a highly activated state of systemic immunity that is characterized by excessive or uncontrolled release of proinflammatory cytokines [5]. It had been proved cytokine storms were associated with the severity in many respiratory diseases, such as bronchiolitis and community-acquired pneumonia [6, 7]. The presence of cytokine storms has also been demonstrated in cases of severe influenza, and plays an important role in the influenza severity [8–10]. Previous studies revealed that excessive cytokines can be detected in influenza patients with poor prognosis [11, 12]. Thus, cytokine levels might be indicative of the prognosis of influenza patients. However, there are very few studies on the correlation between cytokine levels and the prognosis of influenza-associated pneumonia.

In the present study, we have examined the changes in cytokine levels in patients with pneumonia and their association with disease severity. Additionally, and importantly, we have identified biomarkers to predict the prognosis of influenza-associated pneumonia.

## Material and methods

### Study design

This study included patients with influenza who were hospitalized (n = 101) at the First Affiliated Hospital of the Medical College of Zhejiang University between January 2019 and June 2019. The causative microorganisms were identified using polymerase chain reaction tests for respiratory viruses. Diagnosis of mixed or secondary infection was based on Sputum culture. Patients with tuberculosis or non-resected lung cancer, and those who were receiving immunosuppressive therapy or had AIDS were excluded.

The patients were divided into the pneumonia group (n = 64) and the non-pneumonia group (n = 37), based on their imaging reports. Clinical information and laboratory results were collected at the earliest time point after the detection of influenza virus at our hospital. The Curb-65 score was determined in patients with pneumonia. The demographic data, co-morbidities, clinical data, treatment, and final prognosis of these patients are outlined in Table 1. The patients with influenza-associated pneumonia were grouped according to their prognosis: patients who did not require ventilator treatment or extracorporeal membrane oxygenation (ECMO), were not admitted to the Intensive Care Unit (ICU), and did not die within 90 days were considered to have a good prognosis (Group A), while the remaining were considered to have a poor prognosis (Group B). The clinical data for the good and poor prognosis groups are provided in Table 2. This study was approved by the Clinical Research Ethics Committee of the First Affiliated Hospital of the Medical College of Zhejiang University.

**Table 1:** Personal and laboratory findings of 101 cases of with influenza in the non-pneumonia and pneumonia groups

	Patients	Non-pneumonia group (n = 37)	Pneumonia group (n = 64)	P-value
Age (years)	56 (46–67)	56 (37–71.5)	56 (50–67)	0.377
Sex (male)	62 (61.39%)	21 (56.76%)	41 (64.06%)	0.467
Length of stay in hospital (d)	13 (7.5–21)	9 (6.5–17)	15 (9.25–26)	0.003^**^
Underlying disease				
Hypertension	30 (29.7%)	8 (21.62%)	22 (34.38%)	0.177
Diabetes	21 (20.79%)	10 (27.03%)	11 (17.19%)	0.240
Liver diseases	19 (18.81%)	8 (21.62%)	11 (17.19%)	0.580
Cardiovascular diseases	10 (9.9%)	2 (5.41%)	8 (12.5%)	0.250
Respiratory diseases	7 (6.93%)	2 (5.41%)	5 (7.81%)	0.646
Kidney diseases	18 (17.82%)	7 (18.92%)	11 (17.19%)	0.827
Tumor	28 (27.72%)	10 (27.03%)	18 (28.13%)	0.905
Cerebrovascular diseases	6 (5.94%)	0 (0%)	6 (9.38%)	0.138
Respiratory virus				
Influenza A(Undifferentiated)	81 (80.20%)	25 (67.58%)	56 (87.50%)	0.031^*^
Influenza B	20 (19.80%)	12 (32.42%)	8 (12.50%)	0.031^*^
Symptom				
Cough	71 (70.3%)	21 (56.76%)	50 (78.13%)	0.024^*^
Sore throat	7 (6.93%)	4 (10.81%)	3 (4.69%)	0.447
Diarrhea	5 (4.95%)	2 (5.41%)	3 (4.69%)	1.000
Dyspnea	20 (19.87%)	1 (2.70%)	19 (29.70%)	0.001^**^
Chill	10 (9.9%)	3 (8.11%)	7 (10.94%)	0.910
Runny nose	4 (3.96%)	1 (2.70%)	3 (4.69%)	1.000
Muscle soreness	6 (5.94%)	2 (5.41%)	4 (6.25%)	1.000
Laboratory indicators				
WBC (× 10^9^ L^−1^)	5.8 (3.7–7.9)	5.3 (3.45–7.00)	6.15 (4.23–8.28)	0.077
Neutrophil ratio (%)	73.1 (56.3–85.6)	61.3 (48.25–72.80)	78.2 (66.58–88.15)	< 0.001^***^
Lymphocyte ratio (%)	14.7 (8.6–30.6)	26.3 (14.65–38.1)	13.5 (7.45–22.68)	< 0.001^***^
CRP (mg/L)	25.69 (9.10–73.85)	16.85 (7.305–29.485)	33.13 (11.73–102.98)	0.001^**^
ALT (U/L)	22 (13–40)	69 (49–94.5)	66.5 (56.25–84.75)	0.231
AST (U/L)	30 (20–50)	66.8 (59.3–70.8)	59 (50.525–64.025)	0.255
LDH (U/L)	265 (203–354)	61 (35.50–130.50)	60.5 (29.75–172.75)	0.005^**^
CK (U/L)	61 (33–166)	20 (10–39.5)	22 (14–45.5)	0.913
Cr (U/L)	67 (53.5–88)	27 (18–42.5)	32 (20.75–51.25)	0.871
Total protein level (g/L)	61.2 (53.25–67.4)	223 (173–272)	282.5 (225.25–383.75)	< 0.001^***^
Urea nitrogen(mmol/l)	5.78 (3.81–8.78)	4.69 (3.44–7.05)	6.57 (4.22–9.56)	0.023^*^
Treatment				
Antiviral	83 (82.18%)	28 (75.68%)	55 (85.94%)	0.194
Antibiotic	87 (86.14%)	26 (70.27%)	61 (95.31%)	< 0.001^***^
Glucocorticoid	42 (41.58%)	5 (13.51%)	37 (57.81%)	< 0.001^***^
Antifungal	27 (26.73%)	3 (8.11%)	24 (37.5%)	0.001^**^
Prognosis				
Invasive mechanical ventilation	13 (12.87%)	0 (0%)	13 (20.31%)	0.009^**^
ECMO	1 (0.99%)	0 (0%)	1 (1.56%)	1.000
ICU admission	12 (11.88%)	0 (0%)	12 (18.75%)	0.013^**^
90-day mortality	11 (10.89%)	0 (0%)	11 (17.19%)	0.019^**^

WBC, white blood cell; CRP, C-reactive protein; ALT, alanine aminotransferase; AST, aspartate aminotransferase; CK, creatine kinase; LDH, lactate dehydrogenase; Cr, Creatinine; ECMO, Extracorporeal Membrane Oxygenation

Significant differences between non-pneumonia and pneumonia groups are indicated by asterisks

(*: P < 0.05; **: P < 0.01; ***: P < 0.001)

**Table 2:** Personal and laboratory findings of 64 cases of with influenza in the good and poor prognosis groups

	Group A (n = 47)	Group B (n = 17)	P
Age (year)	56 (50–67)	58 (44–66)	0.593
Sex (male)	29 (61.70%)	12 (70.59%)	0.513
Length of stay in hospital(d)	14 (7–18)	26 (18.5–36)	< 0.001^***^
Underlying disease			
Hypertension	17 (36.17%)	5 (29.41%)	0.615
Diabetes	7 (14.89%)	4 (23.53%)	0.665
Liver diseases	10 (21.28%)	1 (5.88%)	0.286
Cardiovascular diseases	5 (10.64%)	3 (17.65%)	0.748
Respiratory diseases	3 (6.38%)	2 (11.76%)	0.856
Kidney diseases	7 (14.89%)	4 (23.53%)	0.665
Tumor	10 (21.28%)	8 (47.06%)	0.087
Cerebrovascular diseases	3 (6.38%)	3 (17.65%)	0.379
Symptom			
Cough	38 (80.85%)	12 (70.59%)	0.593
Sore throat	3 (6.38%)	0 (0%)	0.691
Diarrhea	2 (4.26%)	1 (5.88%)	1.000
Dyspnea	10 (21.28%)	9 (52.94%)	0.014^*^
Chill	7 (14.89%)	0 (0%)	0.218
Runny nose	3 (6.38%)	0 (0%)	0.691
Muscle soreness	4 (8.51%)	0 (0%)	0.511
Viral subtypes			
Influenza A(Undifferentiated)	40 (85.11%)	16 (94.12%)	0.593
Influenza B	7 (14.89%)	1 (5.88%)	0.593
Mixed infection	4 (8.51%)	10 (58.82%)	< 0.001^***^
Pneumonia subtype			
Mixed viral and bacterial	3 (6.38%)	5 (29.41%)	0.042^*^
Primary viral	44 (93.61%)	12 (70.58%)	0.042^*^
Laboratory indicators			
WBC (× 10^9^ L^−1^)	5.7 (4.3–7.4)	8.1 (3.3–14.1)	0.130
Neutrophil ratio (%)	75.2 (62.7–85.7)	86.9 (79.8–93.65)	0.003^***^
Lymphocyte ratio (%)	14.6 (10.3–24.4)	5.9 (3.65–12.2)	0.001^***^
CRP (mg/L)	26.4 (9.9–88.14)	96.02 (30.595–192.97)	0.008^**^
ALT (U/L)	22 (13.75–40)	23 (15.50–70)	0.479
AST (U/L)	32 (20–55)	33.5 (25.5–49.5)	0.794
LDH (U/L)	271 (219–362.5)	339.5 (269.5–493.25)	0.047^*^
CK (U/L)	55.5 (25.75–231.25)	75.5 (37.5–151.25)	0.568
Cr (U/L)	65 (55–85)	69 (58.5–83)	0.627
Total protein level (g/L)	60.2 (52.3–64.1)	54.2 (47.8–63.1)	0.109
Urea nitrogen(mmol/l)	5.81 (4.19–8.63)	9.93 (6.48–14.67)	0.010^*^
Treatment			
Antiviral	38 (80.85%)	17 (100%)	0.052
Antibiotic	44 (93.62%)	17 (100%)	0.691
Glucocorticoid	22 (46.81%)	15 (88.24%)	0.003^***^
Antifungal	12 (25.53%)	12 (70.59%)	0.001^**^

Group A, good prognosis group,patients who did not require ventilator treatment or ECMO, were not admitted to the ICU, and did not die within 90 days. Group B, fatal and serious cases group, patients experienced either ventilator treatment, ECMO, ICU or death

WBC, white blood cell; CRP, C-reactive protein; ALT, alanine aminotransferase; AST, aspartate aminotransferase; CK, creatine kinase; LDH, lactate dehydrogenase; Cr, Creatinine

Significant differences between Group A and Group B are indicated by asterisks

(*: P < 0.05; **: P < 0.01; ***: P < 0.001)

### Measurement of plasma cytokines

The plasma of patients with laboratory-confirmed influenza (n = 101) was collected at the earliest possible time point after influenza virus detection at our hospital. According to the manufacturer’s instructions, the plasma levels of 48 cytokines and chemokines were detected by Bio-Plex pro-human cytokine 48-Plex Panel (Bio-Rad) according to the standard manufacturer’s protocol.

### Statistical analysis

Normally distributed data are presented as the mean and standard deviation, while data with non-normal distribution are presented as the median and interquartile range (IQR). The Mann–Whitney *U*-test was used to analyze the non-normally distributed data. The chi-square test was used to compare categorical data. In the pneumonia group, Spearman rank correlation analysis was used to determine the correlation between plasma cytokine expression levels and the Curb-65 score. Spearman correlation analysis was also used to determine the correlation between the levels of various cytokines. The area under the curve (AUC) based on the receiver operating characteristic (ROC) curve was determined in patients with poor prognosis (Group B). In addition, the comprehensive predictive value of various cytokines for poor prognosis was determined using binary logistic regression analysis. All the statistical tests were conducted using SPSS 23.0 and Graphpad8.0.

## Results

### Demographic and clinical characteristics

Out of the 101 influenza-positive patients who were enrolled in this study, the 37 patients without pneumonia had a median age of 56 years and 56.76% were males (Table 1). In the remaining 64 patients with influenza-associated pneumonia, the median age was 56 years and 64.06% were males. There was no significant difference in age or sex between the two groups (P > 0.05). Several patients had more than one underlying disease, and the highest prevalence rates were observed for hypertension (29.70%), malignant tumor (27.72%), and diabetes (20.79%). There was no significant difference in the prevalence of chronic diseases (P > 0.05). Cough (70.30%) and dyspnea (19.87%) were common clinical manifestations. In the pneumonia group, cough and dyspnea were present in 78.13% (n = 50) and 29.70% (n = 19) of the patients, respectively, while a significantly lower prevalence was observed in the non-pneumonia group (55.81% [n = 24] [P = 0.024] and 2.33% [n = 1] [P = 0.001] for cough and dyspnea respectively).

### Laboratory findings

There was no significant difference in white blood cell count between the two groups (P = 0.08), but the neutrophil percentage in the pneumonia group was significantly higher than that in the non-pneumonia group. Additionally, the lymphocyte percentage in the pneumonia group was significantly lower than that in the non-pneumonia group (P < 0.001). The level of serum C-reactive protein tended to increase in patients with influenza, and it was significantly higher in the pneumonia group than in the non-pneumonia group (P < 0.05). The level of protein in the pneumonia group was significantly lower, while the lactate dehydrogenase level was significantly higher (P < 0 01). There was no significant difference in the alanine aminotransferase, aspartate aminotransferase, or creatinine levels between the two groups.

### Mixed infection

Overall, 14 pneumonia patients (21.88%) had mixed Infection while none in the non-pneumonia group. And the results showed that more mixed infections were found in Group B (P < 0.001) (Table 2). The most common microorganism in mixed or secondary infection was *Acinetobacter baumannii* (50.00%) (Additional file 4: Table S1).

### Treatment and outcomes

Among the patients with influenza-associated pneumonia, 85.94%, 95.31%, 57.81%, and 37.5% received treatment with antivirals, antibiotics, glucocorticoids, and antifungal drugs, respectively, while 75.68%, 70.27%, 13.51%, and 8.11% of the patients in the non-pneumonia group received antivirals, antibiotics, glucocorticoids, and antifungal drugs respectively. There was no significant difference in the use of antiviral drugs, but the use of antibiotics, glucocorticoids, and antifungal drugs in the pneumonia group was significantly higher than that in the non-pneumonia group (P < 0.001). Thirteen patients (20.31%) received ventilator treatment, 12 patients (11.88%) were admitted to the ICU, and 11 patients (10.89%) died within 90 days. All these cases were from the pneumonia group, which means that there was a significant difference between the two groups with regard to these variables (P < 0.05). One patient (0.93%) from the pneumonia group was treated with ECMO, while none of the patients in the non-pneumonia group required ECMO (Table 1). However, the difference was not significant due to the limitation of the small sample size.

### Changes in cytokine levels in patients with influenza

**Fig. 1:**
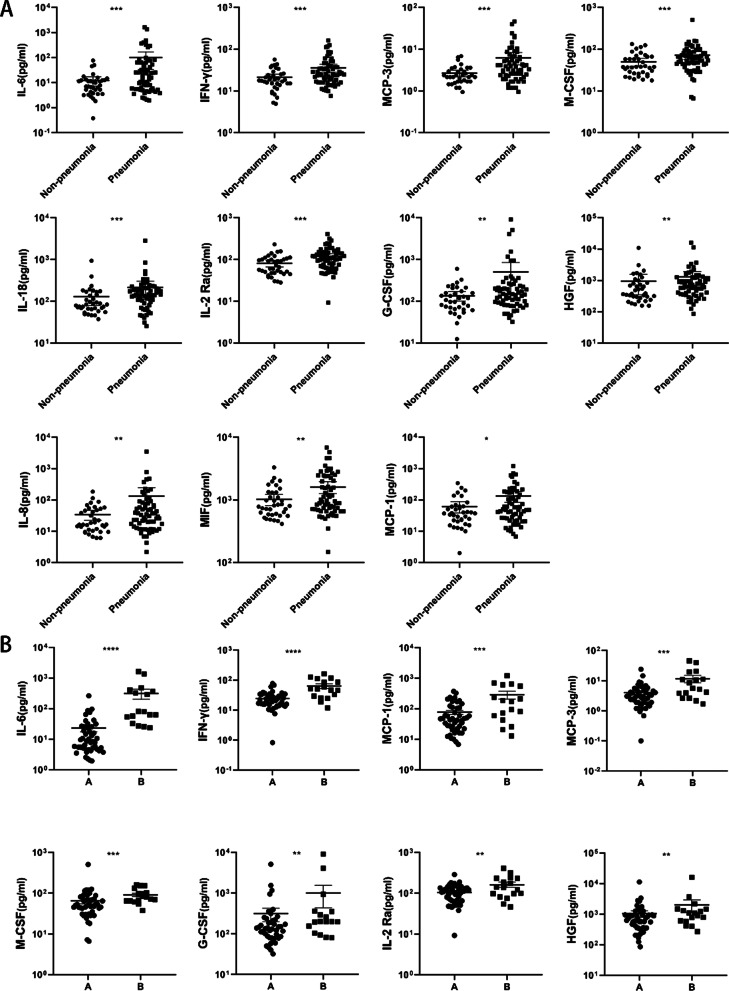
Comparison of the significantly elevated cytokines between different groups. **A** Non-pneumonia and pneumonia (**B**) Pneumonia with a good prognosis (Group A) and with a poor prognosis (Group B). The levels of IL-6, IFN-γ, G-CSF, M-CSF, IL-2 Ra, MCP-1, MCP-3, HGF increased significantly in patients with Pneumonia, especially who with poor prognosis. P values between 0.1 and 0.05, 0.05 and 0.01, 0.001 and 0.001 and less than 0.001 were considered statistically significant and marked as *, **, ***, ****

First, we compared the levels of the 48 analyzed cytokines between the pneumonia group and the non-pneumonia group. The results showed that the levels of 11 cytokines were significantly higher in the pneumonia group than in the non-pneumonia group: interleukin 6 (IL-6), interleukin 18 (IL-18), interferon gamma (IFN-γ), interleukin 8 (IL-8), monocyte chemoattractant protein-1/3 (MCP-1/MCP-3), colony stimulating factor-granulocyte colony stimulating factor (G-CSF), macrophage-stimulating factor (M-CSF), hepatocyte growth factor (HGF), interleukin-2 receptor subunit alpha (IL-2Ra), and macrophage migration inhibitory factor (MIF). We further compared the levels of cytokines between the two influenza-associated pneumonia groups (Group A and Group B). From the 11 cytokines mentioned above, 8 cytokines had significantly higher levels in the patients with a poor prognosis (Group B): IL-6, IFN-γ, G-CSF, M-CSF, MCP-1, MCP-3, IL-2Ra, and HGF (Fig. 1).

### Correlation between cytokine levels and severity of pneumonia

**Fig. 2:**
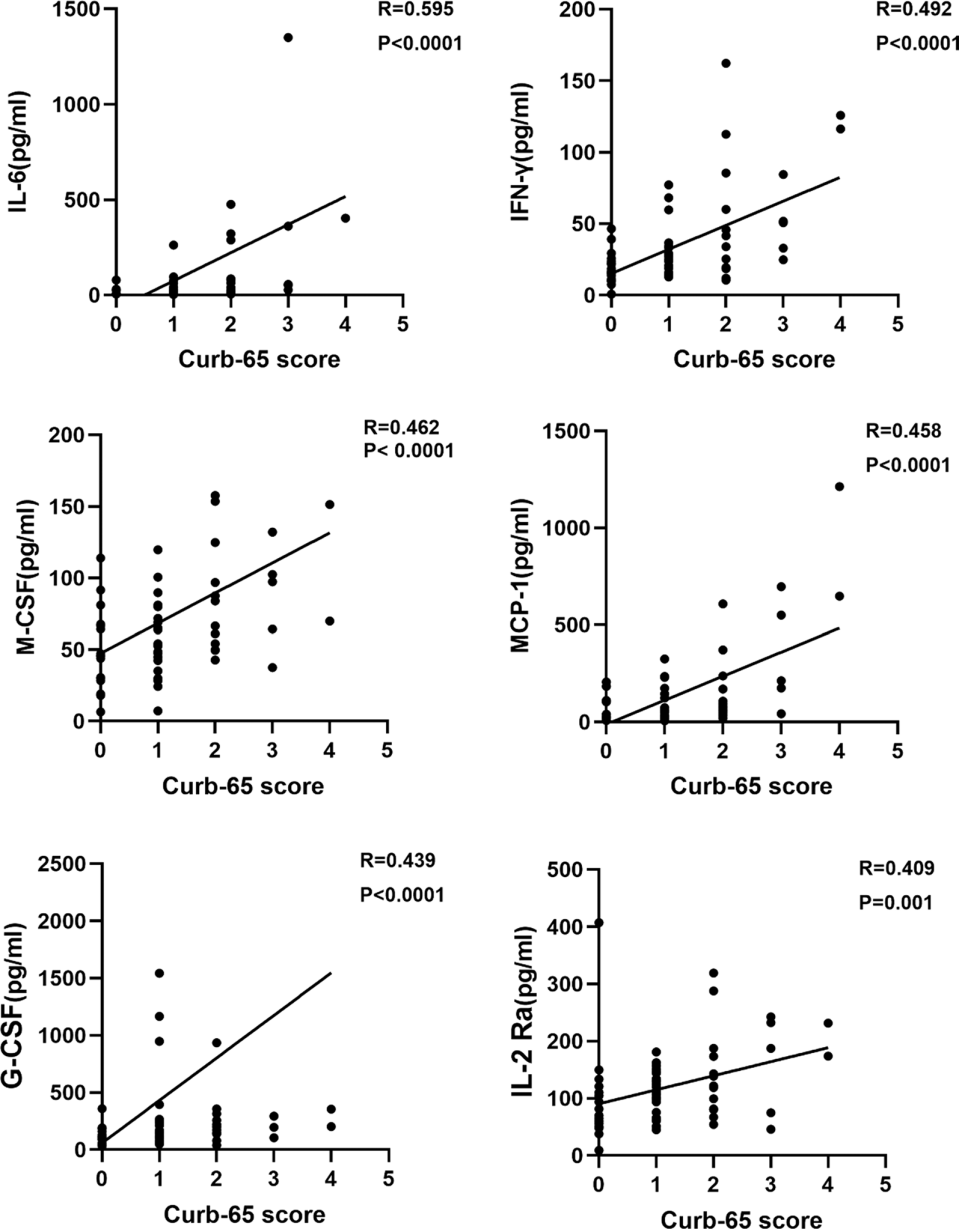
The associations between Curb-65 score and cytokine levels. The expression levels of cytokines measured from plasma samples were from patients with influenza associated pneumonia. The associations were analyzed using Spearman rank correlation analysis. The level of IL-6, IFN-γ, M-CSF, MCP-1, G-CSF and IL-2Ra showed a good and positive correlation with Curb-65 score

The severity of influenza-associated pneumonia was evaluated by the Curb-65 score, which was based on the clinical data collected. The correlation analysis was conducted on the cytokines that were detected at significantly higher levels in Group B. The results showed that there was a strong and positive correlation between IL-6 levels and the Curb-65 score (R = 0.595). Additionally, the levels of IFN-γ (R = 0.492), M-CSF (R = 0.462), MCP-1 (R = 0.458), G-CSF (R = 0.439) and IL-2Ra (R = 0.409) were positively correlated with the Curb-65 score. (Fig. 2). The results of the other cytokines were showed in Additional file 1: Fig. S1.

### Associations between cytokine levels

**Fig. 3:**
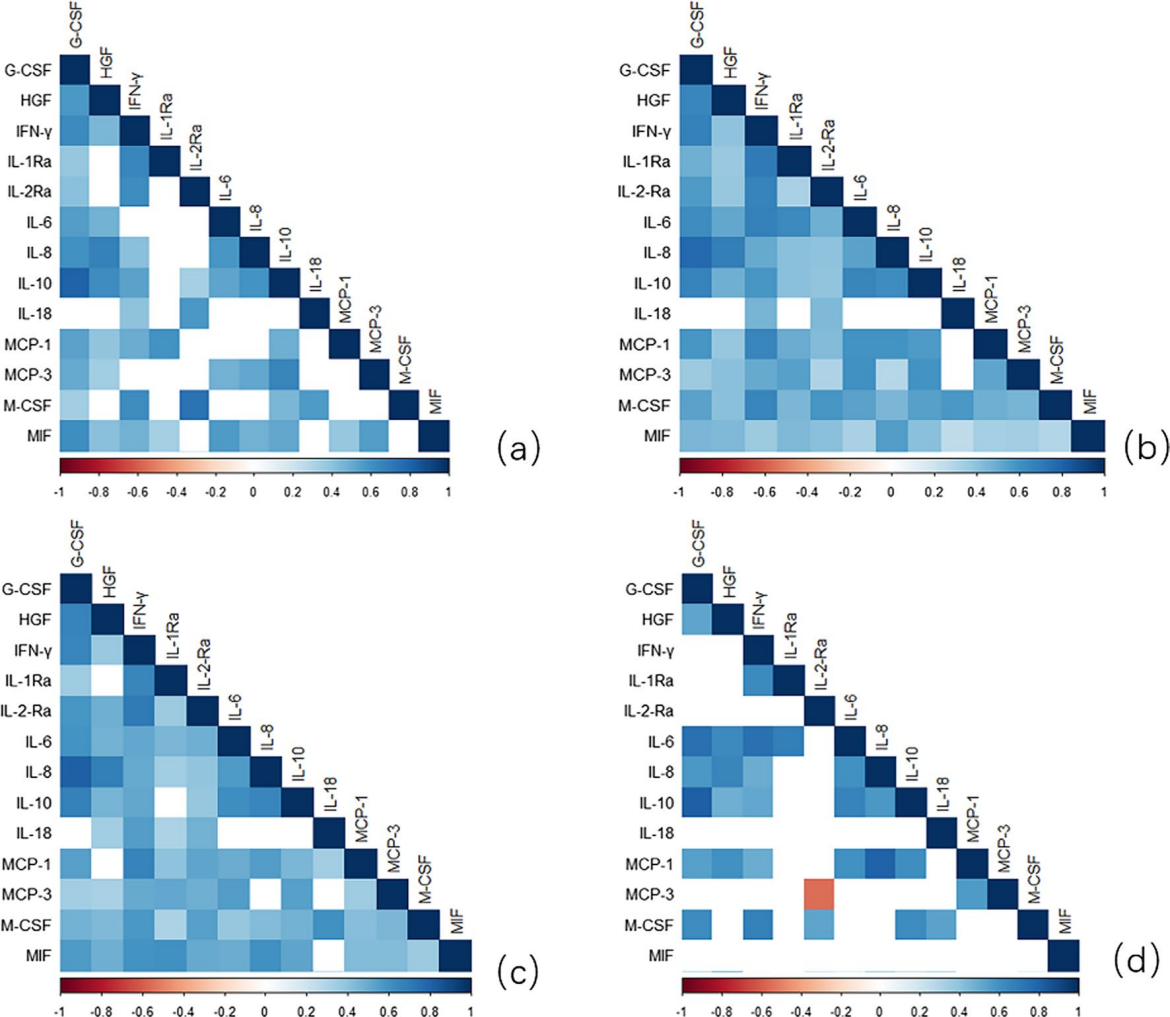
The correlation between cytokines in different states (**a**) Non-pneumonia. **b** Pneumonia. **c** Pneumonia with a good prognosis (Group A). **d** Pneumonia with a poor prognosis (Group B). The results showed that IL-6 expression level was highly and positively correlated with IFN-γ, G-CSF, M-CSF, MCP-1 in different states of disease

Correlation analysis of cytokines was carried out to explore the interaction of cytokines in the disease process. In patients with pneumonia, the IL-6, IFN-γ, G-CSF, M-CSF, IL-1Ra, IL-2Ra, IL-10, HGF, MCP-1, and MCP-3 expression levels showed a strong positive correlation. (Fig. 3b) Further analysis showed that the IL-6 level was strongly correlated with the G-CSF (R = 0.740) and IFN-γ level (R = 0.745) level in Group B. (Fig. 3d) The correlation of IL-6 with G-CSF (R = 0.576) and IFN-γ (R = 0.488) was weaker in Group A (Fig. 3c).

### Role of cytokines in predicting the prognosis of patients with influenza-associated pneumonia

**Fig. 4:**
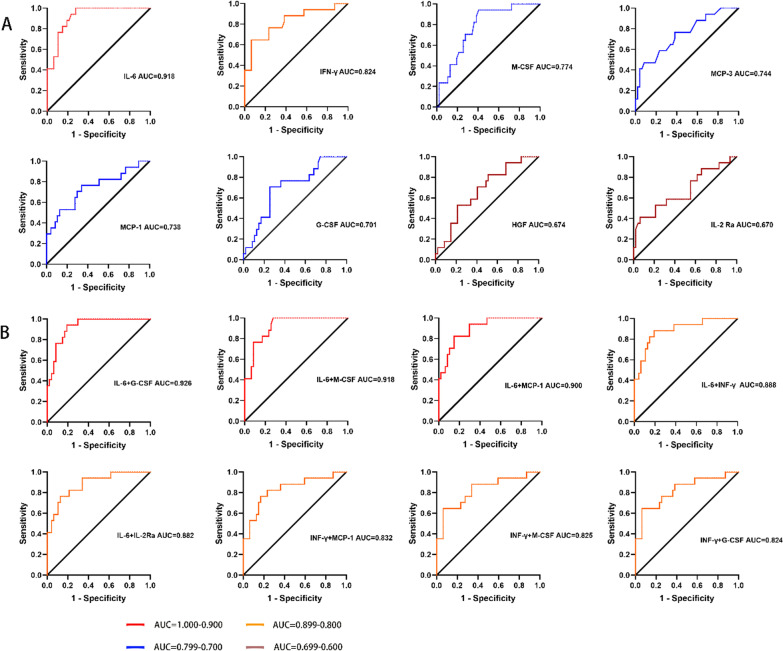
The ROC curve of plasma cytokine levels for patients with different prognosis. The AUC of the ROC curve for IL-6, IFN-γ, G-CSF, M-CSF, IL-2 Ra, MCP-1, MCP-3, HGF was estimated. **A** The ROC curve of each cytokine. **B** The ROC curves of different combination of two cytokines from IL-6, G-CSF, M-CSF, IFN-γ, MCP-1 and IL-2 Ra. All the P values were less than 0.05. ROC, receiver operating characteristic; AUC, area under the curve

The AUC of the ROC curve was 0.918 for IL-6, followed by 0.824 for IFN-γ, 0.774 for M-CSF 0.738 for MCP-1, and 0.701 for G-CSF. The AUC for other cytokines varied from 0.588 to 0.776. (Fig. 4A and Additional file 2: Fig. S2) Then, we tested combinations of different cytokines. The AUC of the ROC curve was 0.926 for IL-6 and G-CSF, 0.918 for IL-6 and M-CSF, 0.900 for IL-6 and MCP-1, and 0.888 for IL-6 and IFN-γ (Fig. 4B). The AUC for other combinations varied from 0.674 to 0.882 (Fig. 4A and Additional file 3: Fig. S3).

## Discussion

This study investigated inflammatory biomarkers that would be useful for predicting the prognosis of patients with influenza who develop pneumonia. Out of 48 cytokines analyzed and compared between pneumonia patients who had a poor prognosis and pneumonia patients who had a good prognosis, the combined predictive value of IL-6 and G-CSF was found to be the most reliable.

In the present study, cough and shortness of breath were the most common symptoms in patients with influenza, and the incidence of dyspnea in patients with pneumonia was significantly higher than that in patients who did not develop pneumonia [13]. These findings are consistent with the results of previous studies (Table 1). Additionally, in the patients with influenza-associated pneumonia, the neutrophil percentage, hypersensitive C-reactive protein level, and lactate dehydrogenase level were significantly higher, while the level of protein was significantly lower, than those in patients without pneumonia (Table 2). This may be caused by the cytokine storms that occur during the course of influenza-associated pneumonia [14, 15]. Fourteen patients with pneumonia were complicated with bacterial or fungal infection. The use of antibiotics, glucocorticoids, and antifungal drugs in patients with influenza-associated pneumonia was significantly higher than that in those without influenza-associated pneumonia. This is probably because patients with pneumonia have more severe disease that necessitates the prevention and control of secondary infections. In our study, the ICU occupancy, mechanical ventilation rate, and mortality in patients with influenza-associated pneumonia patients were significantly higher than those in non-pneumonia patients, and the length of hospital stay of patients with influenza-associated pneumonia was also significantly longer. These findings also corroborate the previous studies [16].

With regard to the cytokine analysis in the present study, we found that the levels of several proinflammatory cytokines (IL-6, IL-18, and IFN-γ), chemotactic proteins (MCP-1/MCP-3 and IL-8), and cell-stimulating factors (G-CSF and M-CSF) were significantly increased in patients with influenza-associated pneumonia (Fig. 1B) Additionally, the neutrophil percentage in patients with influenza-associated pneumonia was significantly higher than that in patients without pneumonia (Table 2) These findings correspond with the immune response pathways that are activated in response to the influenza virus. That is, epithelial cells, lung resident macrophages, and dendritic cells in the lungs produce inflammatory mediators and present the antigen to activate the immune response. Neutrophils are recruited as the first line of defense, and a large number of cytokines are produced to maintain a continuous immune response. Additionally, the bone marrow is mobilized to produce a large number of neutrophils under the stimulation of G-CSF and IL-6 [17, 18]. The neutrophils are activated under the stimulation of IFN-γ [19], and they gather at the inflammatory site under the influence of chemokines (CCL-2/MCP-1, CXCL-8/IL-8, CXCL-9/MIG, and CXCL-10/IP-10) [20, 21]. Thus, the significantly higher levels of cytokines observed in the pneumonia patients in this study may be indicative of a stronger inflammatory immune response.

In our study, the levels of 11 cytokines were found to be significantly elevated in patients with influenza-associated pneumonia. Accordingly, high levels of cytokines have been considered to be related to the high pathogenicity and poor prognosis of influenza for a long time. For example, Ye et al. found that the expression of IL-2, IL-6, IFN-γ, and TNF-α increased significantly in patients with influenza A H1N1 infection [22]. Additionally, Shen et al. found that elevated levels of IL-6, IL-8 and MIP-1 β were associated with a high viral load and poor prognosis in patients with H7N9 influenza infection [23].

In the present study, we found that patients with pneumonia who had a poor prognosis had a significantly higher neutrophil percentage than those who had a good prognosis. Neutrophils can recognize the invasion of pathogens through receptor signaling pathways that include Toll-like receptors, Fc receptors, and G protein-coupled receptors, and release a reticular de-agglutination chromatin, referred to as neutrophil extracellular traps (NETs), that can effectively kill pathogens. However, a couple of studies by Teluguakula et al. have shown that NETs can induce the occurrence of influenza-associated pneumonia [24, 25]. It was reported that a large number of NETs was found in the alveoli, airway, and tissue lesions of patients with influenza-associated pneumonia, and was associated with the occurrence of acute lung injury, acute respiratory distress syndrome, and other complications that led to a poor prognosis [24, 26]. When influenza infection is complicated with bacterial infection, NETs can also promote the production of IFN-γ by dendritic cells under the action of bacterial lipopolysaccharide. In turn, IFN-γ can lead to a poor prognosis by inhibiting bacterial clearance [27]. Accordingly, in our study, we found that the IL-6, M-CSF, G-CSF, and IFN-γ expression levels were positively correlated with the Curb-65 score (Fig. 3). This implies that these cytokines may have a good correlation with the severity of influenza-associated pneumonia.

In this study, we explored the correlation between cytokines. We found that IL-6 was highly correlated to G-CSF in patients with pneumonia (R = 0.613). The correlation was stronger in patients with a poor prognosis (R = 0.740), while it was notably weaker in influenza-associated pneumonia patients with a good prognosis (R = 0.576) (Fig. 3). Similarly, previous studies have also demonstrated that cytokines interact with each other in dynamic ways that involve cytokine receptors and signaling pathways [4]. Additionally, in an inflammatory environment, inflammatory factors, such as antigens and IL-6, can stimulate the production of macrophages, T cells, endothelial cells, and fibroblasts and induce them to secrete G-CSF [28]. Therefore, we speculated that the correlation between IL-6 and G-CSF observed in this study is related to the degree of inflammation.

We further tested whether these cytokines could be used as biomarkers to predict the prognosis of influenza-associated pneumonia. The AUC of ROC for IL-6 was 0.918, and the combination of IL-6 and G-CSF showed a slightly higher value of 0.926. Thus, IL-6 and G-CSF can be used as an excellent combination of biomarkers to predict the prognosis of influenza-related pneumonia (Fig. 4). IL-6 is released by tissue macrophages and is an early and potent inflammatory mediator [29]. In previous studies, IL-6R antagonists were mainly used for the treatment of autoimmune diseases [30–32], but recently, IL-6R-targeting inhibitors were also found to be effective in the treatment of severe and critical COVID-19 [33]. G-CSF is a classical neutrophil-stimulating activator that plays a role in the specific functional responses of neutrophils to the influenza A virus [34]. Blocking the receptor of G-CSF was found to inhibit edema caused by neutrophils through a reduction in neutrophil recruitment, and did not affect the clearance of pathogens [35]. Based on these findings, the potential benefits of IL-6- and G-CSF-targeted therapy in the treatment of influenza-associated pneumonia must be explored in the future.

This study has limitations. First, there was a lack of description of the cytokine dynamics in patients with influenza so that it is impossible to further verify the correlation between cytokine levels and prognosis in course of the disease. Second, as some of patients had received antiviral or antibiotic therapy before admission, we could not rule out the effect of treatment on cytokines. Third, the number of patients is so small that a logistic regression analysis can not be carried to explore independent factors associated with worse outcomes. Finally, the study was just carried from January 2019 and June 2019 in a single center, the results should be verified by more date.

## Conclusion

In summary, in our study, we found that cytokines play an important role in the development of influenza-associated pneumonia. Importantly, our findings showed a significant increase in IL-6 and G-CSF levels in influenza-associated pneumonia patients with a poor prognosis. In the future, the use of IL-6R and G-CSFR antagonists in the treatment of influenza-associated pneumonia patients with high levels of IL-6 and G-CSF needs to be explored.

## Supplementary Information

**Additional file 1**: **Figure S1.** The associations between Curb-65 score and the level of IL-1Ra, SCF, IL-8 MCP-3, IP-10, IL-10 and HGF. The associations were analyzed using Spearman rank correlation analysis.

**Additional file 2: Figure S2. **The ROC curve of IL-1Ra, IL-10, IP-10, SCF, IL-8, G-CSF, IL-18 expression levels upon admission for patients with and without a good prognosis. All the P values were less than 0.05. ROC, receiver operating characteristic; AUC, area under the curve.

**Additional file 3: Figure S3.** The ROC curves of different combination of two cytokines from IL-6, G-CSF, M-CSF, IFN-γ, MCP-1 and IL-2 Ra. All the P values were less than 0.05. ROC, receiver operating characteristic; AUC, area under the curve.

**Additional file 4: Table S1.** Mixed or secondary infection with respiratory virus.

## Data Availability

The datasets used and/or analyzed during the current study are available from the corresponding author on reasonable request.

## Abbreviations

WHO: World Health Organization
ECMO: Extracorporeal membrane oxygenation
IQR: Interquartile range
AUC: Area under the curve
ROC: Receiver operating characteristic
IL-6: Interleukin-6
IL-8: Interleukin 8
IL-18: Interleukin 18
IL-2Ra: Interleukin-2 receptor subunit alpha
MCP-1: Monocyte chemoattractant protein-1
MCP-3: Monocyte chemoattractant protein-3
HGF: Hepatocyte growth factor
IFN-γ: Interferon-γ
MIF: Macrophage migration inhibitory factor
G-CSF: Granulocyte colony-stimulating factor
M-CSF: Monocyte colony-stimulating factor
NETs: Neutrophil extracellular traps
