# Supplementary material for: Interleukin-6 and granulocyte colony-stimulating factor as predictors of the prognosis of influenza-associated pneumonia
Source: BMC Infect Dis. 2022 Apr 6;22:343. doi: 10.1186/s12879-022-07321-6 (PMC8983324; doi:10.1186/s12879-022-07321-6)
Supplement: Supplementary file 1 — Additional file 1: Figure S1. The associations between Curb-65 score and the level of IL-1Ra, SCF, IL-8 MCP-3, IP-10, IL-10 and HGF. The associations were analyzed using Spearman rank correlation analysis. [file 12879_2022_7321_MOESM1_ESM.docx]

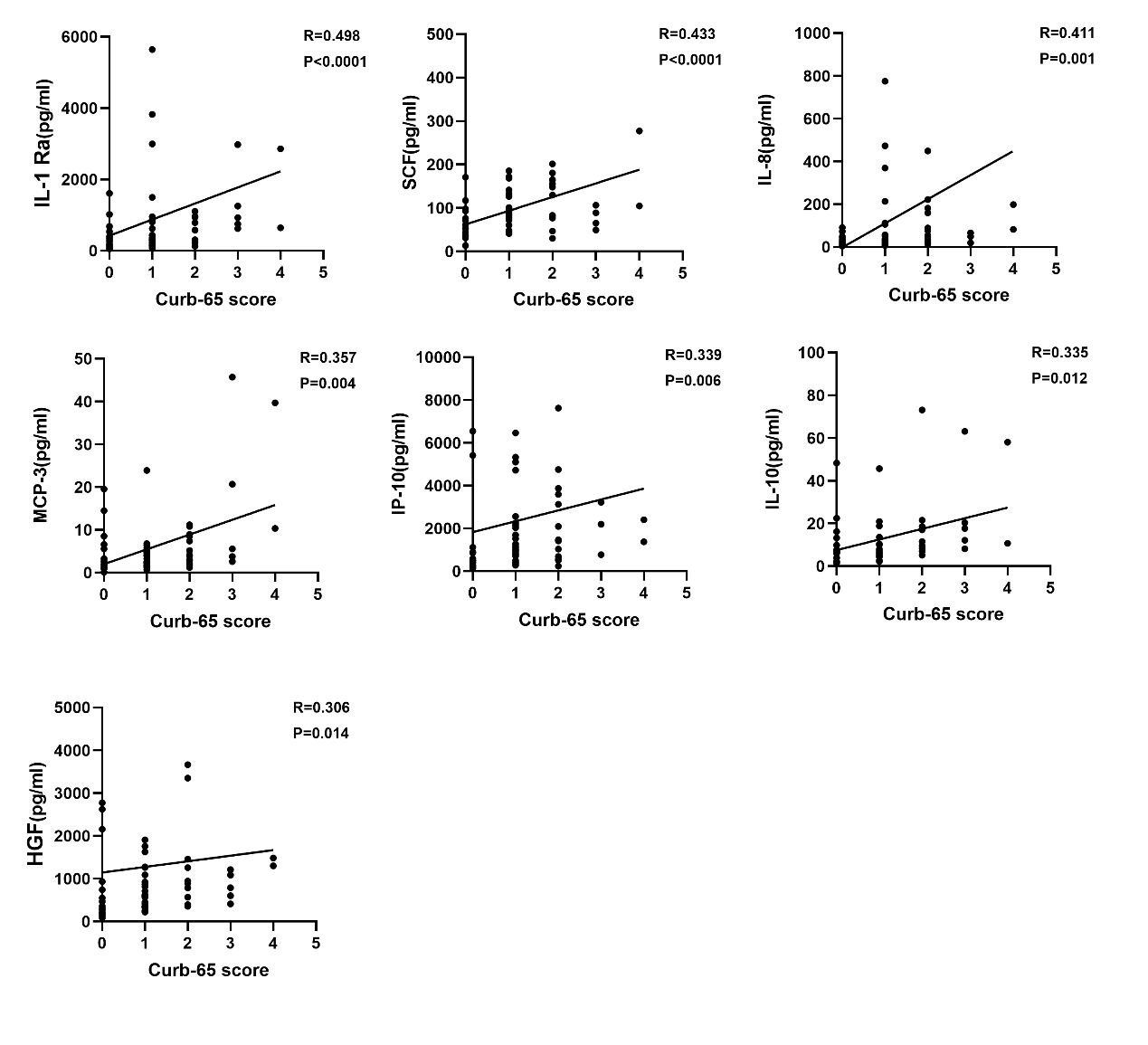


**Figure S1:** The level of IL-1Ra, SCF, IL-8 MCP-3, IP-10, IL-10 and HGF showed a good and positive correlation with Curb-65 score. The associations were analyzed using Spearman rank correlation analysis.
