# Supplementary material for: Interleukin-6 and granulocyte colony-stimulating factor as predictors of the prognosis of influenza-associated pneumonia
Source: BMC Infect Dis. 2022 Apr 6;22:343. doi: 10.1186/s12879-022-07321-6 (PMC8983324; doi:10.1186/s12879-022-07321-6)
Supplement: Supplementary file 2 — Additional file 2: Figure S2. The ROC curve of IL-1Ra, IL-10, IP-10, SCF, IL-8, G-CSF, IL-18 expression levels upon admission for patients with and without a good prognosis. All the P values were less than 0.05. ROC, receiver operating characteristic; AUC, area under the curve. [file 12879_2022_7321_MOESM2_ESM.docx]

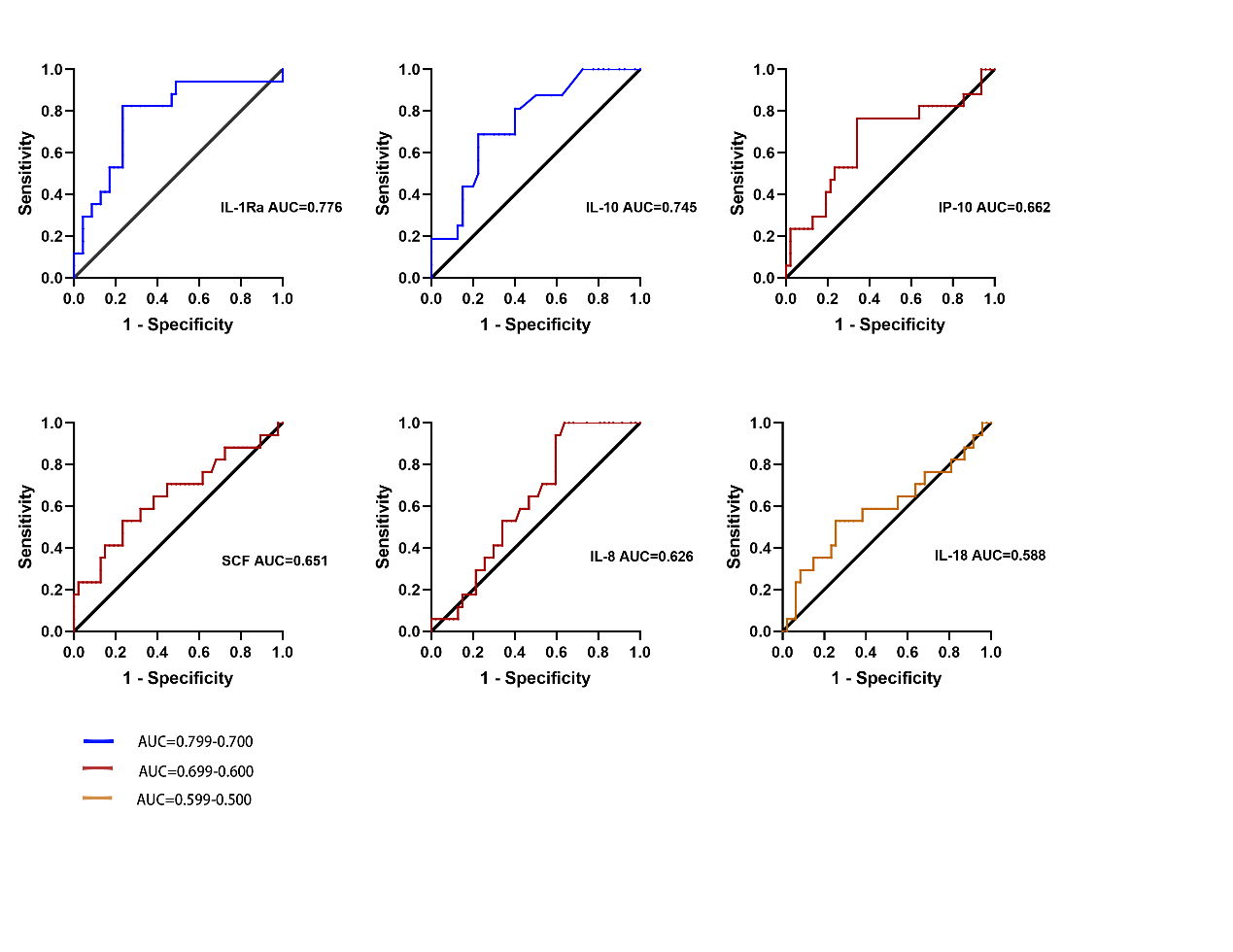


**Figure S2**. The ROC curve of IL-1Ra, IL-10, IP-10, SCF, IL-8, G-CSF, IL-18 expression levels upon admission for patients with and without a good prognosis. All the P values were less than 0.05.

**Abbreviations:** ROC, receiver operating characteristic; AUC, area under the curve
