# Supplementary material for: Interleukin-6 and granulocyte colony-stimulating factor as predictors of the prognosis of influenza-associated pneumonia
Source: BMC Infect Dis. 2022 Apr 6;22:343. doi: 10.1186/s12879-022-07321-6 (PMC8983324; doi:10.1186/s12879-022-07321-6)
Supplement: Supplementary file 3 — Additional file 3: Figure S3. The ROC curves of different combination of two cytokines from IL-6, G-CSF, M-CSF, IFN-γ, MCP-1 and IL-2 Ra. All the P values were less than 0.05. ROC, receiver operating characteristic; AUC, area under the curve. [file 12879_2022_7321_MOESM3_ESM.docx]

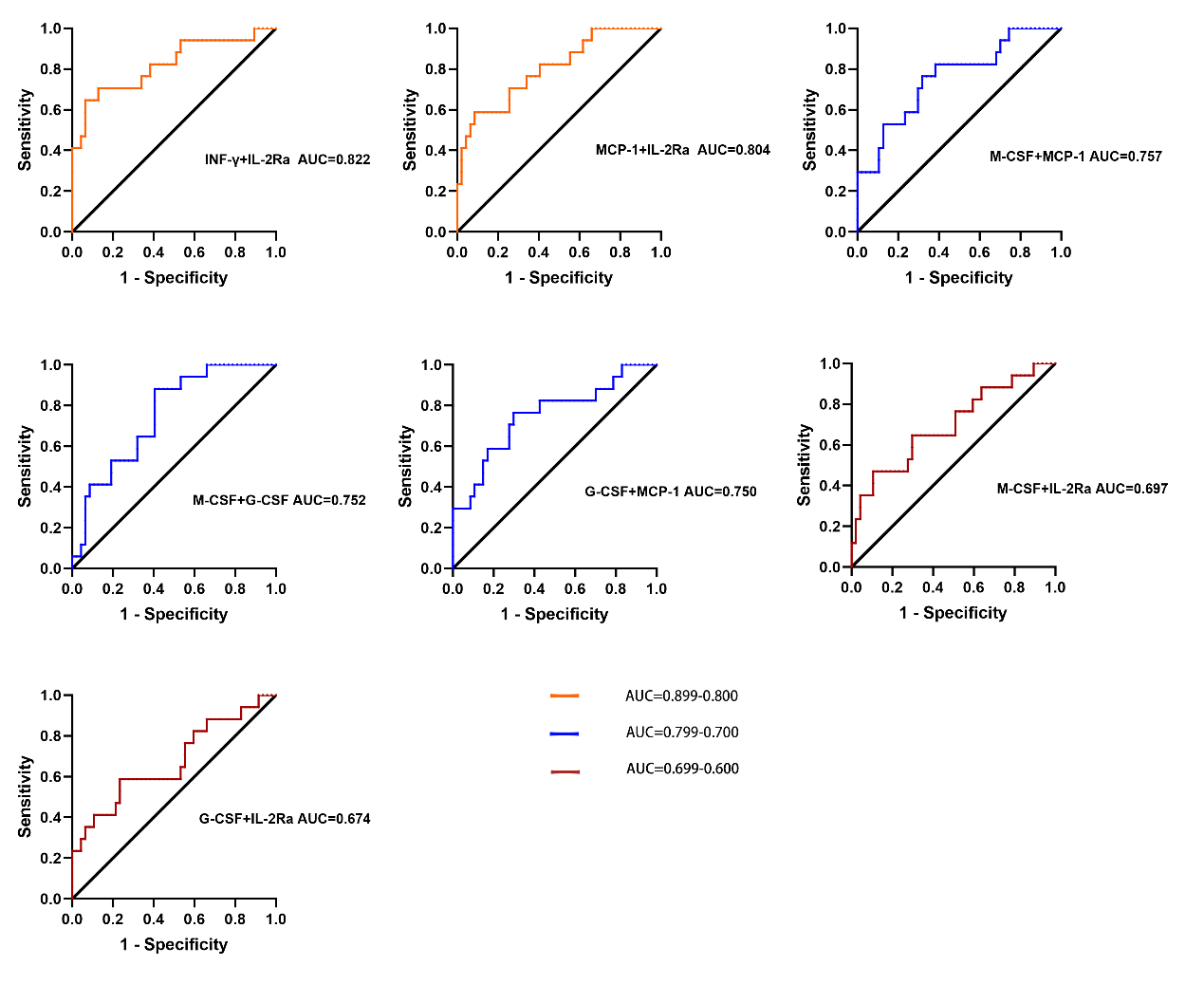


**Figure S3**. The ROC curves of different combination of two cytokines from IL-6, G-CSF, M-CSF, IFN-γ, MCP-1 and IL-2 Ra. All the P values were less than 0.05.

**Abbreviations:** ROC, receiver operating characteristic; AUC, area under the curve
