# Supplementary material for: Interleukin-6 and granulocyte colony-stimulating factor as predictors of the prognosis of influenza-associated pneumonia
Source: BMC Infect Dis. 2022 Apr 6;22:343. doi: 10.1186/s12879-022-07321-6 (PMC8983324; doi:10.1186/s12879-022-07321-6)
Supplement: Supplementary file 4 — Additional file 4: Table S1. Mixed or secondary infection with respiratory virus. [file 12879_2022_7321_MOESM4_ESM.docx]

| Pathogens | Patients (n) |
| --- | --- |
| Two pathogens |  |
| Acinetobacter baumannii+ Klebsiella pneumoniae | 1 |
| Acinetobacter baumannii+ Candida albicans | 1 |
| Oligomonas maltophilia+ Candida albicans | 1 |
| Staphylococcus aureus+ Burkholderia cepacia | 1 |
| One pathogen |  |
| Acinetobacter baumannii | 5 |
| Candida albicans | 1 |
| Klebsiella pneumoniae | 1 |
| Pseudomonas aeruginosa | 1 |
| Staphylococcus haemolyticus | 1 |
| Haemophilus influenzae | 1 |

Table S1 Mixed or secondary infection with respiratory virus.
